# Surgical dental implants in people living with HIV-AIDS

**DOI:** 10.1186/1742-4690-9-S1-P85

**Published:** 2012-05-25

**Authors:** A Sparaco, M Ghezzi, G Donati, K Andriella, A Montebello, C Luraghi, G Romanoni, V Rania

**Affiliations:** 1 Dentistry at Luigi Sacco Hospital, Milan, Italy

## Introduction

The introduction of HAART has considerably improved life expectancy and quality of life of people living with HIV/AIDS (PLWHA).

If in the first years HIV infection was considered an absolute contraindication to implantology, it is now possible to employ implants positioning which allows a more complete functional and aesthetic rehabilitation of the oral cavity also in these patients; howewer there is still fear and prejudice in using this technique in PLWHA.

We present the experience of Department of Dentistry of Luigi Sacco Hospital of Milan in over twenty years of implant surgery to evaluate the possibility of using implantology techniques in PLWHA without exposing them to greater risks of developing infections both during and after surgical intervention.

## Materials and methods

The study considers a consecutive series of 21 HIV-positive patients (for a total 80 implants) and 91 HIV-negative patients as control group (for a total 245 implants), treated in the dental clinic of the Luigi SaccoHospitalfrom 1998 to december 2011.

The pre-surgery phase included a collection of anamnesis, clinical examination data, diagnostic radiology evaluation and assessment of blood tests.

We used the surgical technique of submerged fixture implants and mobile and fixed prostheses for the final prosthetic rehabilitation.

## Results

Several failures occurred in both groups and were attributable to local factors related to receiving bone or to exceedingly invasive surgical techniques.

We detected no lesions in the oral cavity in these subjects concomitant with the plants loss nor changes in their overall health conditions.

## Conclusions

Patients enrolled in this study presented both functional and aesthetic dental problems.

The comparison between the success/failure rates in the two groups shows that implant surgery can be employed without risk for the patient and with success rate comparable to the general population, nevertheless it is important to assess the level of immune competence of the patient.

Finally, the prosthetic rehabilitation of the oral cavity, in addition to the clear local benefit, has an important psychological effect on patients and on your quality of life.

